# Facile Fabrication of Low‐Impedance, Highly Conformal Epidermal Electrodes Based on Laser‐Induced Graphene–Silver Nanocomposites

**DOI:** 10.1002/advs.76138

**Published:** 2026-06-15

**Authors:** Jiuqiang Li, Senhao Zhang, Kai Guo, Donghai Qiu, Juzhong Zhang, Hongbo Yang, Huanyu Cheng

**Affiliations:** ^1^ Suzhou Institute of Biomedical Engineering and Technology Chinese Academy of Science Suzhou P. R. China; ^2^ Department of Engineering Science and Mechanics The Pennsylvania State University University Park Pennsylvania USA; ^3^ School of Biomedical Engineering (Suzhou) Division of Life Sciences and Medicine University of Science and Technology of China Hefei P. R. China

**Keywords:** dry epidermal electrodes, facile fabrication, human–machine interface, laser‐induced graphene nanocomposites, long‐term stability

## Abstract

Long‐term and high‐fidelity electrophysiological (EP) signal monitoring with conformal epidermal electrodes with low contact impedance and robustness against motion artifacts is of great significance for health monitoring and human–machine interfaces. However, the low‐cost yet scalable fabrication of large‐area electrodes and arrays with these desired properties remains challenging. Here, a facile strategy is reported to fabricate large‐area, highly conformal, and low‐impedance epidermal electrodes based on highly porous and conductive laser‐induced graphene (LIG) nanocomposites. The patterned superhydrophilic LIG allows the self‐selective wetting of silver ionic solutions into the porous structure, followed by reduction to form LIG–Ag nanocomposites with significantly reduced sheet resistance (from 50 to 0.06 Ω/sq) and also the skin contact impedance. Subsequent transfer of the nanocomposites onto an ultrathin (13 µm) styrene‐ethylene‐butylene‐styrene (SEBS) substrate results in durable, highly conformal, and low‐impedance epidermal electrodes even after storage for over 8 months. The resulting electrodes demonstrate superior signal‐to‐noise ratio (SNR) and resistance against motion artifacts for EP signal monitoring. The twelve‐lead ECG monitoring and biopotential‐based control of a robotic hand demonstrate the practical utility of these electrodes for clinical health monitoring and human–machine interactions.

## Graphical abstract

A facile laser‐induced graphene–silver nanocomposite strategy enables ultrathin epidermal electrodes with low impedance, high conductivity, and excellent mechanical robustness. The porous LIG–Ag conductive network mechanically interlocked with SEBS provides stable, motion‐resistant biopotential monitoring, enabling high‐fidelity ECG, EMG, and EEG recording for long‐term wearable bioelectronics and human–machine interfaces.

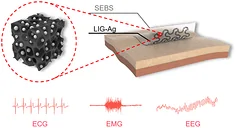

## 1 Introduction

Epidermal electrophysiological (EP) signals, commonly represented by electrocardiogram (ECG), electromyography (EMG), electrooculography (EOG), and electroencephalography (EEG), contain rich information about body activities and health status [1, 2, 3, 4, 5, 6, 7]. The high‐precision and long‐term monitoring of EP signals contributes to clinical diagnosis, fitness tracking, and human–machine interfaces [8, 9, 10, 11]. Epidermal electrodes, with a comparable Young's modulus and stretchability to human skin, can form conformal contact with the skin for non‐invasive and real‐time monitoring of EP signals [12, 13].

In conventional clinical practice, Ag/AgCl gel electrodes remain the gold standard due to their low initial contact impedance and reliable signal acquisition. However, they suffer from several well‐recognized limitations, particularly in long‐term and wearable applications. These include: (i) dehydration and degradation of the conductive gel over time, leading to increased impedance and signal deterioration; (ii) poor mechanical compliance with soft and dynamically deforming skin, resulting in motion artifacts; and (iii) potential skin irritation during prolonged use [14]. To address these challenges, stretchable dry electrodes [15] based on serpentine‐patterned metal thin films and conductive polymers [16] have emerged as a promising alternative for long‐term, high‐precision EP signal monitoring. However, complex structured designs are required to impart stretchability to intrinsically non‐stretchable metals (despite superior electrical conductivity), which typically involves complex and costly processes (e.g., photolithography) with low material utilization [17, 18]. Conductive polymers offer improved softness and processability, yet their long‐term electromechanical stability, environmental robustness, and interfacial adhesion to soft elastomers remain limited under repeated deformation and perspiration conditions [19, 20]. In addition, direct printing approaches generally deposit conductive materials onto the surface of elastomers, resulting in weak interfacial adhesion with soft substrates [17]. By contrast, infiltrating an elastomer precursor into a porous conductive material, followed by curing and transfer, forms a continuous and mechanically interlocked conductive network, providing excellent mechanical stability and enhanced resistance to motion artifacts for reliable long‐term EP signal recording.

With high electrical conductivity, flexibility, and chemical stability, ultra‐thin graphene has been widely explored as epidermal electrodes [21, 22]. Compared with traditional graphene preparation methods such as chemical vapor deposition, laser‐induced graphene (LIG) [23, 24] features large‐area, patterned, porous structures directly formed on commercially available polyimide (PI) films or other carbon‐containing materials. To form conformal contact with the skin, LIG typically prepared on PI or polyetherimide needs to be transferred onto stretchable substrates (e.g., PDMS and Ecoflex) [23]. However, LIG with a sheet resistance of 30–100 Ω/sq further increases after transfer to result in high contact impedance at the electrode/skin interface and low signal‐to‐noise ratio (SNR) in the obtained EP signals [25, 26, 27]. The LIG‐based soft electrodes are also associated with a sparse conductive network and high and unstable impedance to give reduced resistance against motion artifacts. Efforts to address this issue have led to the modification of LIG with conductive polymers (e.g., poly(ethylene dioxythiophene): poly(styrene sulfonate) (PEDOT: PSS) [22]) or metals (e.g., copper [28]) with high electrical conductivity, but the improvements in conductivity, even with those complex preparation processes, are limited. Therefore, it is highly desirable to improve both the conductivity and the stability of the conductive network in LIG, thereby obtaining LIG‐based low‐impedance epidermal electrodes.

To address these challenges, we propose a materials–structure–fabrication co‐design strategy based on a LIG–Ag/SEBS hybrid system. Specifically, this study reports a facile strategy to prepare high‐performance and durable LIG‐based epidermal electrodes by leveraging the superhydrophilic LIG on near‐hydrophobic PI for selective wetting of silver ionic solution into the porous LIG network. After thermal reduction, LIG–Ag nanocomposites are formed, where Ag nanoparticles and their aggregates are uniformly incorporated within the three‐dimensional porous scaffold, creating a dense and interconnected conductive network. This hybrid structure is subsequently transferred onto an ultrathin styrene‐ethylene‐butylene‐styrene (SEBS) substrate, where the conductive network is mechanically interlocked with the substrate, resulting in a compliant yet robust LIG–Ag/SEBS epidermal electrode system. By simultaneously optimizing conductivity, mechanical compliance, interfacial stability, and manufacturability, the proposed platform overcomes the limitations of conventional patterned metals, conductive polymers, and previously reported LIG‐based electrodes. The incorporation of Ag nanoparticles creates a dense and redundant conductive network within the porous LIG, placing the composite well above the percolation threshold. Meanwhile, the ultrathin SEBS substrate enhances conformal adhesion to the skin and significantly reduces skin–electrode contact impedance.

As a result, the LIG–Ag/SEBS electrodes exhibit low contact impedance, high signal fidelity, and excellent long‐term stability, maintaining high‐quality ECG signals during continuous 5‐day wear and even after more than 8 months of storage, thereby overcoming the intrinsic limitations of gel‐based electrodes. This mechanically robust network preserves electrical continuity under deformation, enabling stable, long‐term EP signal recording with enhanced SNR and strong resistance against motion artifacts. The proof‐of‐concept demonstrations of large‐area twelve‐lead ECG recording and human–machine interface further highlight the versatility of the LIG–Ag/SEBS electrodes, providing a feasible route for future clinical health monitoring and advanced human–machine interactions (HMI).

## 2 Results and Discussion

### 2.1 Preparation of LIG–Ag/SEBS Skin‐Conformal Electrodes

**FIGURE 1:**
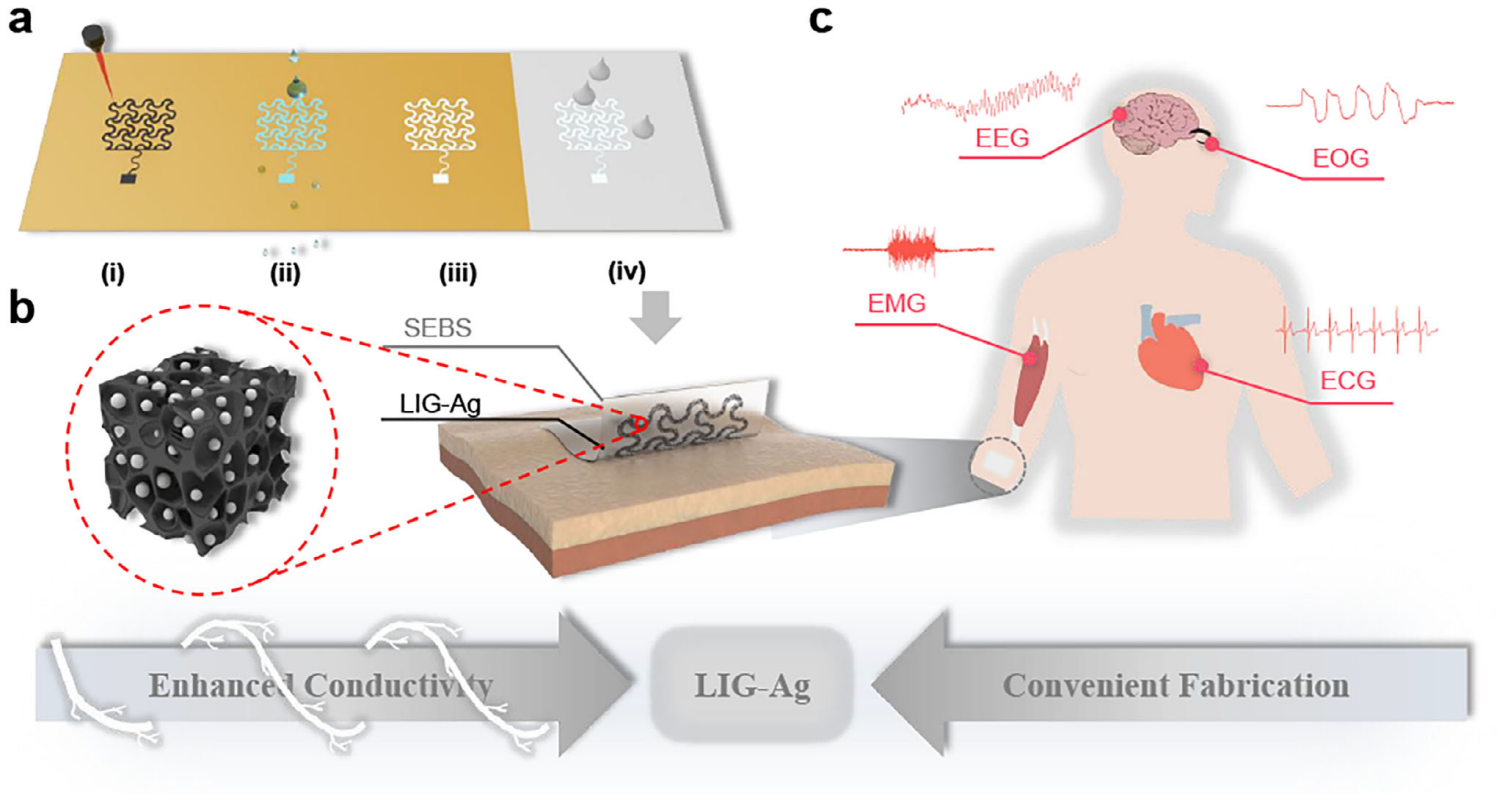
Fabrication process and application of ultrathin epidermal electrodes based on laser‐induced graphene (LIG)–silver (Ag)/SEBS. (a) Schematic illustrating the fabrication process of LIG–Ag/SEBS electrodes: (i) LIG patterning; (ii) selective Ag ionic solution wetting; (iii) thermal reduction to LIG–Ag; (iv) elastomer‐assisted PI etching and electrode transfer. (b) Schematic of the LIG–Ag/SEBS epidermal electrodes in conformal contact with the skin, with the Ag nanoparticles embedded within the porous LIG structure shown in the inset. (c) Illustration of epidermal electrophysiological (EP) signal monitoring, including electrocardiograms (ECG), electromyograms (EMG), electrooculograms (EOG), and electroencephalograms (EEG).

The fabrication process of the epidermal electrode begins with direct patterning of the electrodes by irradiating and patterning commercial polyimide (PI) films with a 10.6 µm CO_2_ infrared laser, converting the exposed regions into porous graphene (Figure 1a‐i). The electrode pattern is designed with an open‐mesh, serpentine layout to enhance the stretchability and level of comfort. Subsequently, filling the porous structure of LIG with Ag nanoparticles improves the electrical conductivity of the electrodes. This is achieved by selectively wetting the LIG patterns with a reactive silver ionic solution (Figure 1a‐ii), enabled by the wettability contrast between hydrophilic LIG and near‐hydrophobic PI (Figure S1). The reactive silver ionic solution is prepared using a modified Tollens’ process (Figure S2), as described in detail in the Experimental Section [29, 30]. After annealing at 90°C, Ag nanoparticles are formed in situ within the porous structure of LIG, yielding LIG–Ag nanocomposites (Figure 1a‐iii).

Finally, the LIG–Ag nanocomposite is transferred onto a SEBS substrate (Figure 1a‐iv), yielding a LIG–Ag/SEBS epidermal electrode. This process involves spin‐coating a thin layer of SEBS solution in toluene onto the LIG–Ag composite on PI [21]. Compared to conventional silicone elastomers (e.g., PDMS or Ecoflex) that are typically applied via casting and mechanical peeling, the solution‐processable SEBS can deeply infiltrate the porous LIG–Ag network during spin‐coating, forming robust mechanical interlocking after solvent evaporation. In addition, *π–π* interactions between the styrene blocks and graphene, together with interfacial hydrogen bonding, further enhance adhesion at the interface. The spin‐coating process also forms an ultrathin substrate (∼10 µm), resulting in low bending stiffness and superior conformability compared to bulk‐cast silicone films, which are generally much thicker. After solvent evaporation, the PI substrate is chemically etched to realize a stress‐free transfer, eliminating significant stress concentrations at the delamination front that would otherwise damage the fragile percolating network. This ensures the preservation of structural integrity and electrical performance, resulting in highly conformal, low‐impedance, and durable epidermal electrodes. The resulting electrodes prepared by simple and facile fabrication maintain excellent conformal contact with the skin, even under skin deformation (Figure 1b), allowing for continuous acquisition of high‐precision EP signals (Figure 1c).

It is worth noting that while Ag/AgCl modification is a widely adopted strategy in traditional bioelectrodes to improve interfacial stability and reduce polarization, especially in gel‐assisted systems [31], current LIG–Ag electrodes are specifically designed for dry, gel‐free epidermal interfaces. In such conditions, signal stability primarily relies on intimate conformal contact and low, stable contact impedance rather than electrochemical equilibrium at an electrolyte‐mediated interface. Introducing an AgCl layer, which has lower intrinsic conductivity than metallic Ag, may increase baseline impedance and partially disrupt the highly conductive percolation network within the porous LIG–Ag framework. Moreover, in the absence of a stable electrolyte layer, the Ag/AgCl interface may not operate under ideal reversible conditions, potentially leading to interfacial instability under mechanical deformation. Nevertheless, Ag/AgCl modification could be advantageous in applications involving sweat or hydrogel‐assisted interfaces, and thus represents a promising direction for future optimization of application‐specific performance.

### 2.2 Characterization of LIG–Ag/SEBS Skin‐Conformal Electrodes

**FIGURE 2:**
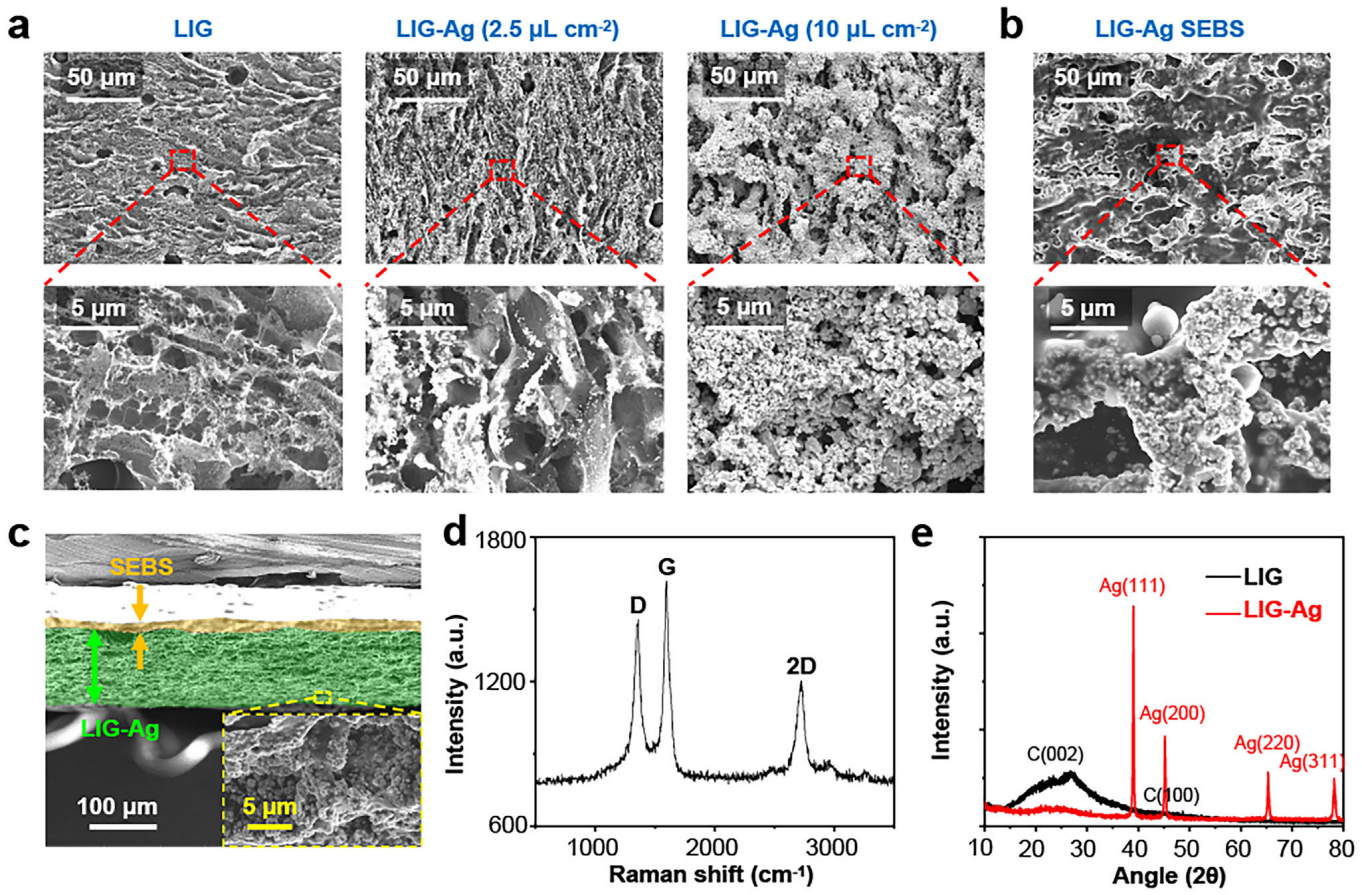
Morphological and material characterizations of LIG–Ag/SEBS epidermal electrodes. (a) Scanning electron microscope (SEM) images of pristine LIG and LIG–Ag with different silver deposition densities. (b) Top‐view SEM image of LIG–Ag transferred onto SEBS, with high‐magnification images shown at the bottom. (c) Cross‐sectional SEM image of the LIG–Ag/SEBS electrode. (d) Raman spectrum of LIG. (e) X‐ray diffraction (XRD) patterns of LIG (black) and LIG–Ag (red).

The morphology/structure and material characterizations confirm the formation of the LIG–Ag nanocomposites. The color of LIG first exhibits a distinct change from black to silver after silver deposition (Figure S3). SEM images reveal the microstructure changes of LIG with increasing silver deposition density (defined as the volume of silver precursor solution deposited per unit area of LIG, µL cm^−2^) (Figure 2a). Owing to the rapid gas release during laser scribing, LIG exhibits a porous morphology with distinct parallel stripe patterns corresponding to the scanning intervals of the pulsed laser. This porous structure increases the surface area and facilitates the penetration of the silver ionic solution. Moreover, SEM images after silver deposition clearly show widely distributed Ag nanoparticles, and the surface of LIG becomes uniformly covered by Ag nanoparticles with increasing Ag deposition, confirming the effectiveness of the selective silver deposition strategy.

To quantitatively characterize the morphology of the deposited Ag, particle size analysis is performed based on high‐magnification SEM images (Figure S4). The results reveal a broad but strongly right‐skewed distribution, with the majority of particles in the nanoscale range of 20–80 nm and a median size of ∼50 nm (Figure S5). Larger features (100 nm to submicron scale) formed due to particle aggregation during growth are also observed, demonstrating the formation of Ag nanoparticles and their aggregates. High‐resolution SEM images further show that the Ag deposits consist of discrete particles with well‐defined faceted geometries, indicating crystalline growth rather than an amorphous structure. Notably, clear particle boundaries and interparticle voids are observed, confirming that the deposited Ag forms a percolated nanoparticle network rather than a continuous film.

Top‐view SEM images of LIG–Ag transferred onto SEBS further reveal that SEBS fully infiltrates into the pores of the LIG–Ag layer (Figure 2b). This strong interfacial integration with a mechanical interlocking structure ensures minimal structure change and conductivity loss during the transfer process and mechanical deformation during on‐body measurements. The cross‐sectional SEM image confirms a total thickness of 133 µm for the resulting electrode (Figure 2c). The image is false‐colored to distinguish different layers, revealing an SEBS layer with a thickness of ∼13 µm and an LIG–Ag nanocomposite layer of ∼120 µm. In addition, a magnified view shows that Ag nanoparticles penetrate into the porous LIG to a depth of approximately 120 µm, indicating the formation of a dense and redundant conductive network throughout the porous LIG framework.

The Raman spectrum of LIG features three characteristic peaks (Figure 2d): the D band (1359 cm^−1^), G band (1596 cm^−1^), and 2D band (2723 cm^−1^), confirming the successful formation of graphene. In addition, comparative Raman spectra of pristine LIG and LIG‐Ag (Figure S6) reveal a pronounced increase in the overall Raman intensity after Ag deposition. This enhancement is attributed to surface‐enhanced Raman scattering (SERS) induced by the Ag nanostructures integrated within the porous LIG framework. The hierarchical architecture, consisting of a three‐dimensional graphene network decorated with Ag nanoparticles, is expected to generate localized electromagnetic “hot spots,” leading to amplified Raman scattering. These results further confirm the successful integration of Ag within the LIG structure and highlight the potential of the LIG‐Ag system as a multifunctional platform, particularly for SERS‐based sensing applications.

The X‐ray diffraction (XRD) patterns of pristine LIG before silver deposition exhibit a strong diffraction peak at 2θ = 26.8° (Figure 2e), corresponding to the (002) plane of graphitic materials. After silver deposition, five additional peaks appear at 2θ = 38.99°, 45.17°, 65.27°, and 78.10°, corresponding to the (111), (200), (220), and (311) planes of face‐centered cubic silver, respectively. These results confirm the successful formation of LIG–Ag nanocomposites.

Subsequently, the evaluation of the electrical properties of the electrodes reveals the decreased sheet resistance as the silver deposition density is increased (Figure S7). Pristine LIG exhibits a sheet resistance of approximately 50 Ω/sq, consistent with previous reports [24, 29]. With the increasing silver deposition density, the sheet resistance of the resulting nanocomposite gradually decreases to reach 0.1 Ω/sq at a deposition density of 10 µL/cm^2^. Further increasing the silver deposition density does not lead to significant conductivity improvement, indicating the optimal deposition density of 10 µL/cm^2^. After transfer onto SEBS, the sheet resistance only increases by approximately two to three times compared to that before transfer, which is sufficiently small when compared with the contact impedance for EP measurements. In addition, the relative resistance change under stretching (Figure S8) shows a clear decreasing trend with increasing Ag deposition density. This behavior can be well explained by a percolation network model, as schematically illustrated in Figure S9. In pristine LIG, electrical transport relies on sparse and fragile conductive pathways that are easily disrupted by microcrack formation and gap opening under tensile strain, leading to abrupt increases in resistance. By contrast, the incorporation of Ag nanoparticles creates a dense and highly interconnected three‐dimensional percolation network within the porous LIG framework. Upon stretching, multiple redundant conductive pathways remain electrically connected even if local junctions are disrupted, thereby suppressing network disconnection and reducing strain‐induced resistance variation.

To further substantiate the mechanical robustness and evaluate the long‐term reliability of the electrodes, comprehensive electromechanical tests are conducted. Comparative stress–strain measurements reveal that the LIG–Ag/SEBS composite electrode maintains a remarkable elongation at break of ∼460%, compared to ∼565% for the pristine SEBS film (Figure S10), which still far exceeds the typical deformation range of human skin. Furthermore, loading–unloading tests demonstrate negligible resistance hysteresis at 30% tensile strain (Figure S11), while only moderate hysteresis is observed at 50% strain. In addition, stable and highly repeatable resistance responses are maintained over 1000 stretching cycles at 30% tensile strain (Figure S12), indicating excellent reversibility and durability under dynamic deformation. The patterning resolution of LIG–Ag/SEBS electrodes is evaluated using linear arrays with varying line widths and spacings (Figure S13), indicating a minimum achievable feature line width of 150 µm with a spacing of 150 µm.

### 2.3 Long‐Term, Motion Artifact‐Free ECG Signal Monitoring Based on LIG–Ag/SEBS Skin‐Conformal Electrodes

**FIGURE 3:**
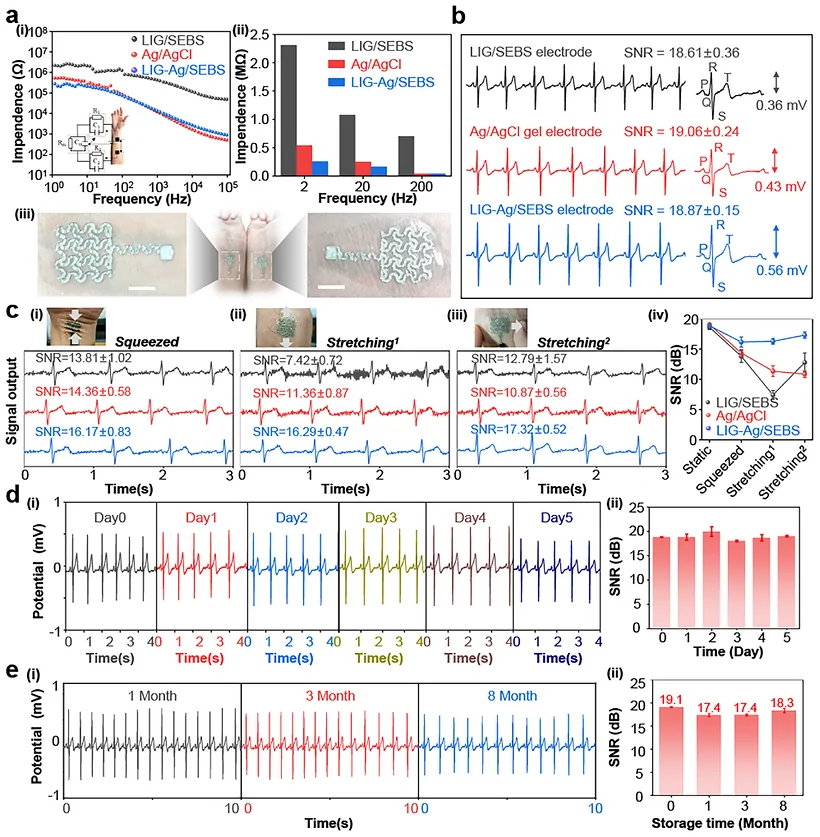
Low‐impedance, motion artifact‐free, and long‐term stable ECG recording using LIG–Ag/SEBS epidermal electrodes. (a) (i) Skin–electrode interfacial contact impedance comparison of LIG, LIG–Ag/SEBS, and Ag/AgCl electrodes, and (ii) contact impedance values at representative frequencies, along with (iii) the optical images of LIG–Ag/SEBS electrodes on skin before (left) and after (right) compression to highlight the conformal contact even during motion. (b) Representative ECG signals and corresponding signal‐to‐noise ratio (SNR) recorded using LIG/SEBS, Ag/AgCl gel, and LIG–Ag/SEBS electrodes. (c) Comparison in ECG signals measured by LIG/SEBS, Ag/AgCl gel, and LIG–Ag/SEBS electrodes under various motion‐induced artifacts, together with a comparison of the corresponding SNR. (d) (i) Five‐day continuous ECG monitoring using LIG–Ag/SEBS electrodes and (ii) the corresponding SNR over time. (e) (i) ECG signals and (ii) SNR after long‐term storage of the fabricated electrodes.

The equivalent circuit model of dry electrodes on skin indicates that the contact impedance of the electrode on skin decreases with increasing frequency (Figure 3a‐i). A lower and more stable contact impedance, especially at low frequency ranges, is beneficial to achieve a higher SNR in EP signal recording, even during motion. The performance evaluation of the epidermal electrodes for EP signal monitoring is carried out by measuring the skin–electrode interfacial contact impedance from three different electrodes (i.e., Ag/AgCl, LIG/SEBS, and LIG–Ag/SEBS electrodes) attached to two identical locations on the forearm with a separation distance of 10 cm. The LIG–Ag/SEBS electrodes exhibit lower contact impedance than that from Ag/AgCl electrodes at frequencies below 300 Hz (Figure 3a‐ii), which corresponds to the main frequency range of EP signals. These results indicate that the LIG–Ag/SEBS electrodes are more suitable for high‐quality EP signal monitoring than LIG/SEBS and Ag/AgCl electrodes.

Subsequently, three LIG–Ag/SEBS electrodes placed at the left leg and the inner wrists of the left and right arms (according to the Einthoven triangle principle) allow for ECG monitoring (Figure 3a‐iii). Enhanced adhesion of the ultrathin electrodes to the skin is achieved with a breathable liquid bandage (Nexcare, 3 m) (Movie S1). The adhesion of the liquid bandage is measured by performing a 90° peel test on the forearm, giving an adhesion of approximately 24 N/m (Figure S14). The effect of the liquid bandage on signal quality is further evaluated by comparing ECG recordings acquired with and without the liquid bandage. As shown in Figure S15, the application of the liquid bandage significantly improves the amplitude and stability of the ECG signals, resulting in a substantially higher SNR. This enhancement is attributed to the improved conformal contact and suppressed micro‐slippage at the skin–electrode interface, a mechanical stabilization mechanism that is generally beneficial for most dry electrode systems.

However, the high SNR achieved in practical wearable applications relies on a synergistic interplay between this interfacial adhesion and the intrinsic electrical properties of the electrode. Before motion‐induced artifacts are applied, the ECG signals recorded using Ag/AgCl, LIG/SEBS, and LIG–Ag/SEBS electrodes all exhibit clear characteristic P waves, QRS complexes, and T waves (Figure 3b), corresponding to atrial depolarization, ventricular depolarization, and ventricular repolarization, respectively. Notably, the LIG–Ag/SEBS electrodes demonstrate higher signal amplitude with comparable SNR than the other two electrodes, indicating high quality of the recorded ECG signals [32]. In this static regime, the liquid bandage primarily functions to ensure a reliable physical interface, demonstrating that once stable contact is established, multiple systems can achieve high‐quality signal acquisition.

In contrast, the intrinsic advantages of the LIG–Ag/SEBS electrode become particularly evident under dynamic conditions. The impact of skin deformation on ECG signal quality is further evaluated under compression, longitudinal stretching, and transverse stretching (Figure 3c). The commercial Ag/AgCl electrodes exhibit significant signal degradation under longitudinal and transverse stretching, indicating insufficient and unstable contact with the skin under these deformation conditions. Moreover, the LIG/SEBS electrodes also show pronounced signal degradation under all three deformation modes, primarily due to their higher and less stable contact impedance in the low‐frequency range relevant for EP signal acquisition. In addition, electrical transport in pristine LIG relies on sparse and discontinuous conductive pathways. Under mechanical deformation, especially tensile strain, microcrack formation and inter‐flake gap opening readily disrupt these pathways, resulting in abrupt increases in resistance and amplified impedance fluctuations. Together, these effects lead to degraded signal amplitude and increased susceptibility to motion artifacts. In contrast, the LIG–Ag/SEBS electrodes maintain excellent signal quality under deformation, which can be attributed to their high conformability and stable, low interfacial contact impedance. More specifically, the incorporation of Ag nanoparticles forms a dense and highly interconnected three‐dimensional percolation network within the porous LIG framework. During tensile deformation, even if local conductive junctions are disrupted, multiple redundant pathways remain electrically connected, effectively preventing network disconnection and suppressing strain‐induced resistance fluctuations. Together, these features give rise to a mechanically robust and failure‐tolerant conductive architecture, thereby ensuring the superior resistance of LIG–Ag/SEBS electrodes to motion artifacts and enabling stable EP signal acquisition under dynamic skin deformation. Notably, even under a 30% tensile strain, the electrodes maintain high‐fidelity ECG recordings with an excellent SNR of 18.46 ± 0.16 (Figure S16).

Therefore, the liquid bandage and the electrode serve complementary but distinct roles: the former ensures stable mechanical contact at the interface, while the highly conductive and resilient percolation network of the latter governs the electrical stability and resistance to motion‐induced artifacts. Only through this synergy can high and stable SNR be achieved in realistic, dynamic wearable scenarios. It is worth noting that achieving this robust architecture requires a careful balance between electrical conductivity and mechanical stability. While increasing the Ag precursor density initially improves conductivity, excessive Ag loading (e.g., 20 µL/cm^2^) tends to block the porous LIG network, which severely hinders the deep infiltration of the SEBS elastomer. This compromised infiltration weakens the interfacial mechanical interlocking, making the electrode more susceptible to delamination and motion‐induced noise under dynamic conditions (Figure S17). Therefore, 10 µL/cm^2^ is selected as the optimal deposition density to establish a saturated conductive network without sacrificing essential mechanical reliability.

Moreover, after 24 h of continuous wear, the LIG–Ag/SEBS electrode exhibits minimal skin irritation and superior skin compatibility, whereas the commercial Ag/AgCl gel electrode induces noticeable erythema upon removal (Figure S18). In addition, a five‐day ECG recording from the continuously worn LIG–Ag/SEBS electrodes also shows maintained high quality (Figure 3d). Furthermore, to simulate a warm and highly humid environment, the electrodes are stored at 30°C and 90% relative humidity (RH, maintained by a saturated KNO_3_ solution) for 7 days (Figure S19). After continuous exposure to this environment, the relative resistance change of the LIG–Ag/SEBS electrodes remains at a low level (5.4%), and the ECG signal waveform and SNR exhibit almost no significant attenuation (18.78 to 18.66). Taken together with the almost unchanged ECG signals (with comparable SNR) even after eight months of storage under ambient laboratory conditions (∼25°C, ∼40%–60% RH) in Figure 3e, the results confirm the long‐term environmental stability, reliable EP signal monitoring capability, and skin compatibility of the LIG–Ag/SEBS electrodes.

These results, together with the fact that the LIG–Ag network is structurally encapsulated within the SEBS matrix to minimize nanoparticle exposure and potential release, and considering the barrier function of the stratum corneum, suggest favorable short‐term epidermal compatibility. Nevertheless, comprehensive biocompatibility evaluations following established standards (e.g., ISO 10993) will be required for future clinical translation.

**FIGURE 4:**
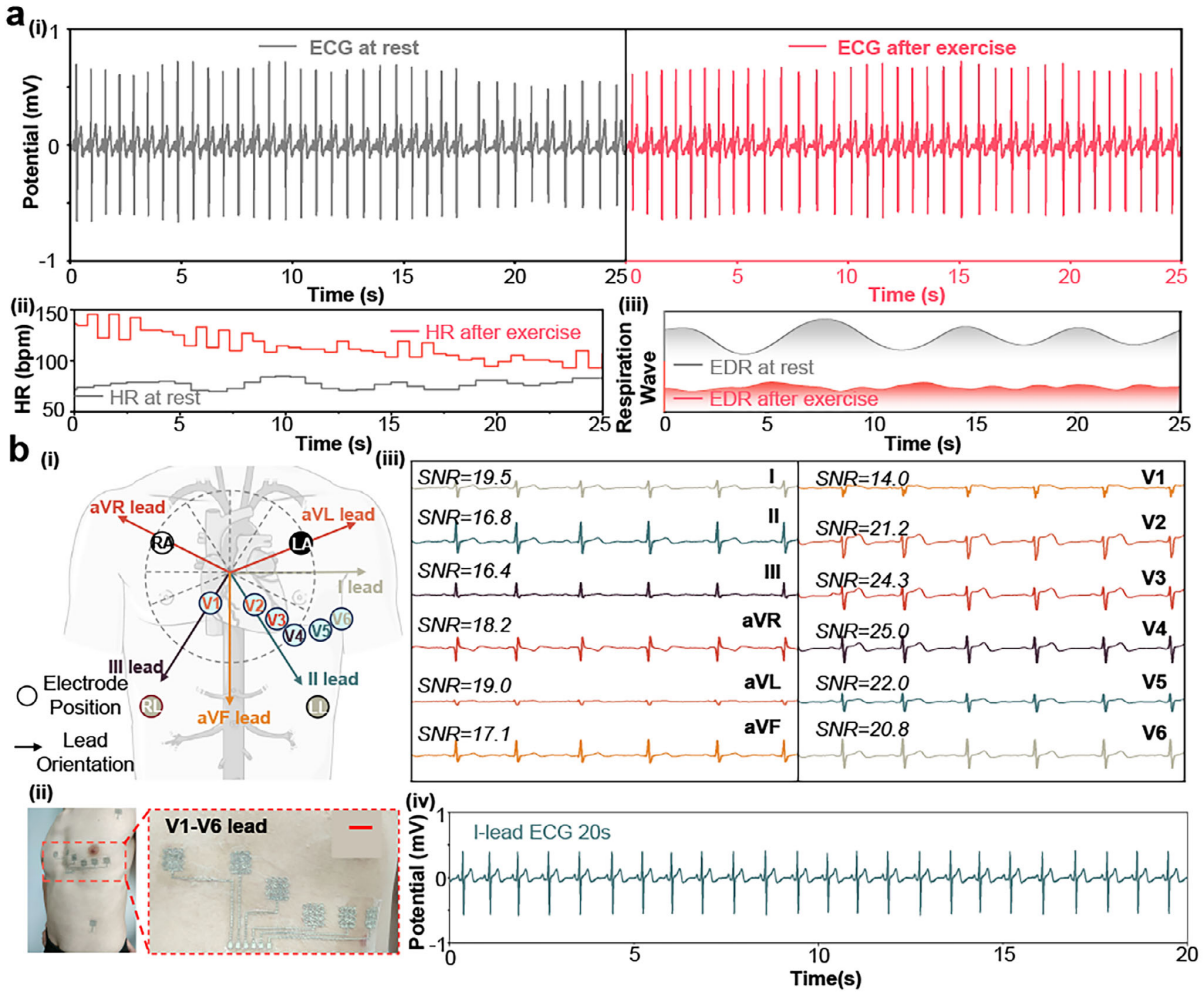
Dynamic and multi‐channel ECG recording based on epidermal LIG–Ag/SEBS electrodes. (a) (i) ECG at rest (left) and after exercise (right), along with (ii) extracted heart rate and (iii) respiration signals. (b) Large‐area 12‐lead ECG recording from a patterned LIG–Ag/SEBS electrode array: (i) schematic and (ii) optical images showing the electrode placement on the chest, (iii) representative waveforms from limb leads (I, II, III, aVR, aVL, and aVF) and precordial leads (V1–V6), compared with (iv) a representative single‐lead ECG signal.

Based on ECG signals recorded from a human subject before and after exercise, the corresponding heart rate and respiratory signals can be extracted (Figure 4a), using an ECG‐derived respiration (EDR) algorithm based on respiration‐induced modulation of R‐wave amplitudes. Briefly, beat‐to‐beat R‐peak amplitudes are interpolated and low‐pass filtered to obtain the respiratory waveform for estimating the respiratory rate from the dominant frequency component. While the heart rate is increased from 80 beats per minute at rest to 120 beats per minute after exercise, the respiration rate is also increased from 9.9 to 17.7 breaths per minute, accompanied by a decrease in amplitude by about 4.6 times. These results demonstrate that the highly conformal LIG–Ag/SEBS electrodes provide a convenient and reliable platform for ECG monitoring (along with heart and respiration rates) before and after physical activities [33].

The standard 12‐lead ECG system is a critical clinical tool for comprehensive cardiac assessment, as it can record cardiac electrical activity using 10 electrodes placed at specific locations on the limbs and chest (Figure 4b‐i). Among these electrodes, the precordial leads (V1–V6) are positioned around the chest, making them suitable for integration into a single large‐area electrode array. Owing to their excellent patternability and low interconnect resistance (<2 Ω), LIG–Ag/SEBS epidermal electrodes are well suited for individual customization of a large‐area, high‐performance precordial electrode array, with minimal biopotential signal loss during signal transmission and human motions. As a result, a chest electrode array with six precordial leads (V1–V6) and interconnect lines is designed (Figure 4b‐ii) by following the slightly modified fabrication process (Figure S20). Together with four single‐lead electrodes placed on the limbs, the precordial electrode array with integrated six electrodes of the same pattern as the single‐lead electrodes and the interconnect lines (width of 600 µm) forms a complete 12‐lead ECG system. With the custom‐built data acquisition and processing circuit board based on an ADS1298 test board (Figure S21), the 12‐lead ECG signals from a healthy male adult can be recorded and analyzed. The ECG signals from all channels exhibit clear P waves, QRS complexes, and T waves, confirming the high quality and stability of the recorded signals (Figure 4b‐iii). These results support the great potential of LIG–Ag/SEBS epidermal electrodes for large‐area electronic integration and multi‐channel clinical monitoring [34].

Compared to conventional flexible electrode fabrication methods that rely heavily on cost‐prohibitive processes such as photolithography, vacuum deposition, and cleanroom facilities, the LIG–Ag/SEBS strategy offers compelling advantages in both cost‐effectiveness and scalability. As detailed in Table S1, the total material and operational cost for fabricating a large‐area 12‐lead ECG electrode array is remarkably low (approximately $1.60). This is enabled by a completely maskless, vacuum‐free, and digitally programmable laser direct‐writing process.

Furthermore, by leveraging the pronounced wettability contrast between superhydrophilic LIG and near‐hydrophobic PI, the silver precursor undergoes self‐selective wetting to enable precise metallization without the need for shadow masks. To facilitate scalability toward roll‐to‐roll (R2R) manufacturing, the lab‐scale spin‐coating step can be readily replaced by scalable techniques such as slot‐die coating, blade coating (doctor blading), or spray coating. Since all processing steps—from laser scribing to solution‐based deposition and elastomer coating—are performed under ambient conditions, this strategy is inherently compatible with continuous R2R manufacturing, providing a practical pathway for the large‐scale production of epidermal bioelectronics.

### 2.4 EMG, EOG, and EEG Signals Monitoring and Robotic Hand Manipulation

**FIGURE 5:**
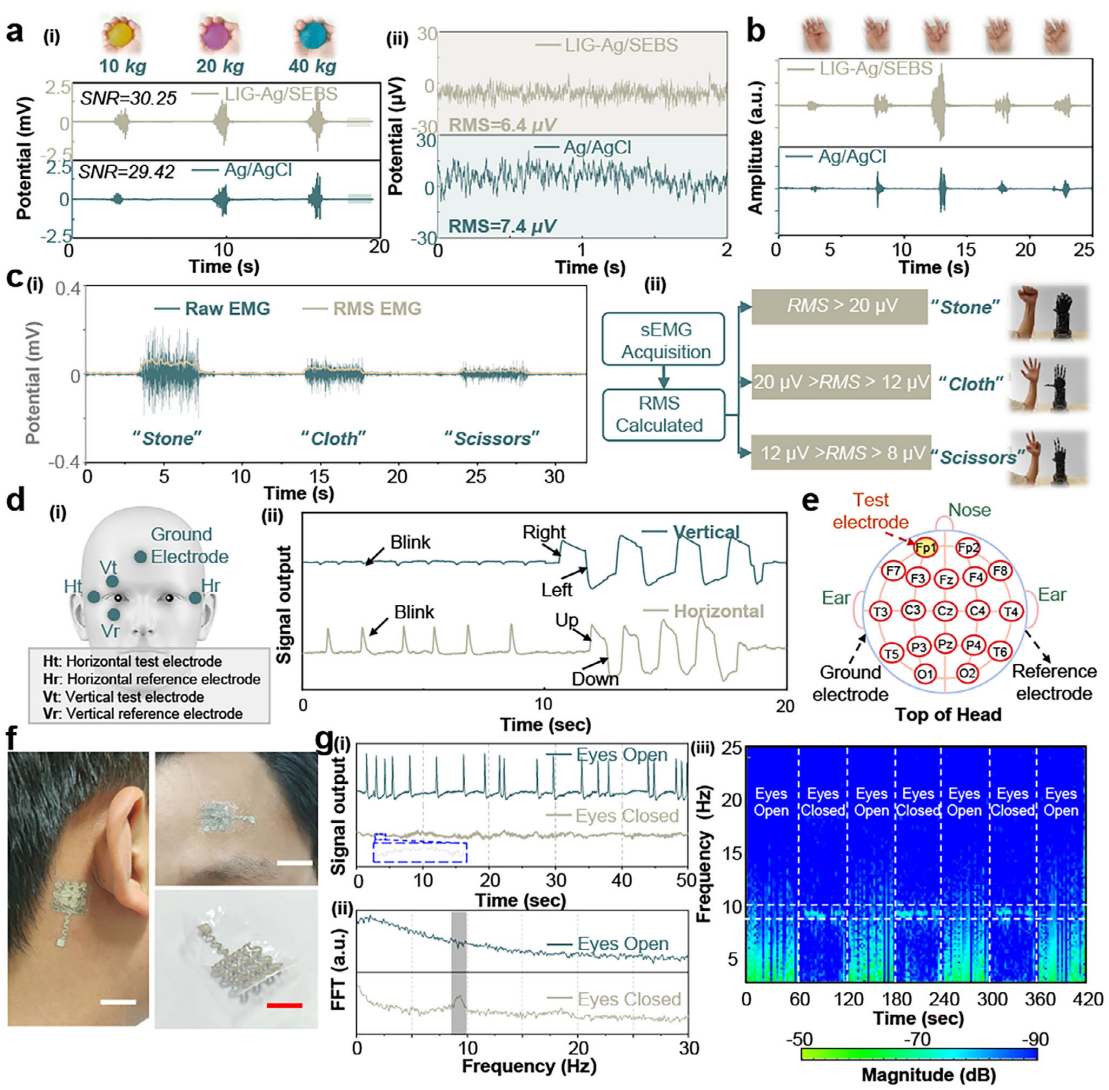
EMG, EOG, and EEG signals monitoring and robotic hand manipulation. (a) (i) sEMG signals recorded using LIG–Ag/SEBS and Ag/AgCl electrodes during gripping of elastic balls at different forces, together with the corresponding SNRs; (ii) baseline sEMG signals comparison between LIG–Ag/SEBS and Ag/AgCl electrodes. (b) sEMG signals generated by different finger flexions measured by LIG–Ag/SEBS and Ag/AgCl electrodes. (c) (i) Representative raw and RMS‐processed sEMG signals recorded during hand gestures (“rock”, “paper”, and “scissors”) and (ii) schematic of RMS‐based sEMG signal processing and threshold classification for hand‐gesture recognition. (d) (i) Schematic showing the location of electrodes for measuring vertical and horizontal EOG signals; (ii) Vertical and horizontal EOG signals measured by LIG–Ag/SEBS electrode. (e) Schematic showing the location of electrodes for measuring EEG signals. (f) Photographs of conformal LIG–Ag/SEBS electrodes for measuring EEG signals. (g) (i) Representative EEG signals during eyes‐open and eyes‐closed states; (ii) Fast Fourier transform spectra; and (iii) time–frequency spectrogram during cyclic eye opening and closing.

LIG–Ag/SEBS electrodes can also be used to record surface electromyography (sEMG) signals associated with action potentials generated by muscle fibers. After applying two electrodes on the forearm as working electrodes with one placed behind the elbow joint as a reference electrode, the EMG signals are recorded while holding elastic balls with different gripping forces (10, 20, and 40 kg) (Figure 5a‐i), demonstrating increased EMG signal amplitude with increasing gripping force. Compared with the signals obtained using Ag/AgCl electrodes, the EMG signals measured by LIG–Ag/SEBS electrodes feature smaller baseline fluctuations and larger amplitudes. Specifically, the baseline noise of 6.4 µV from the LIG–Ag/SEBS electrodes is smaller than that of 7.4 µV from the Ag/AgCl electrodes (Figure 5a‐ii), whereas the SNR of 30.25 from the LIG–Ag/SEBS electrodes is higher than that of 29.42 from the Ag/AgCl electrodes. The LIG–Ag/SEBS electrodes on the forearm can also detect low‐amplitude sEMG signals induced by individual finger movements (Figure 5b), with amplitudes consistently higher than those recorded using Ag/AgCl electrodes. These results highlight the potential of LIG–Ag/SEBS electrodes for high‐precision HMI applications in healthcare and rehabilitation. All in‐study evaluations are conducted under identical conditions (same subject, placement, motion modes, and acquisition parameters), ensuring a fair assessment of intrinsic electrode performance. Compared with other epidermal electrodes fabricated using different methods and materials previously reported, the LIG–Ag/SEBS electrodes demonstrate clear advantages, featuring facile fabrication, high conductivity, long‐term durability, and motion artifact‐free EP signal monitoring with high SNR and lower contact impedance (Table S2). Notably, the reported SNR values are obtained under different testing conditions and therefore should be interpreted as indicative of general performance trends rather than directly comparable metrics.

After integrating the LIG–Ag/SEBS electrodes with a wireless EMG acquisition system based on an AD8221ARMZ chip, the root mean square (RMS) values of the EMG signals can be calculated on the host computer in real time. Owing to the reliable recognition of low‐amplitude EMG signals, multiple different gestures could be accurately distinguished and used for real‐time robotic hand control. The three representative gestures—rock, paper, and scissors—are accurately identified based on the RMS amplitude (higher than 20 µV, between 20 and 12 µV, and between 12 and 8 µV for rock, paper, and scissors) for HMI (Figure 5c). After gesture recognition, the corresponding control commands are transmitted from the host computer to the robotic hand for real‐time control (Movie S2). Applying the LIG–Ag/SEBS electrodes around the eyes (Figure 5d‐i) effectively captures eye movements (e.g., blinking and vertical/horizontal motions) during EOG recording. The EOG signals exhibit periodic fluctuations that clearly correspond to eye movements (Figure 5d‐ii), demonstrating the suitability of the LIG–Ag/SEBS electrodes for EOG recording.

Placing electrodes on the head according to the international 10–20 system (Figure 5e) further allows recording of high‐quality EEG with low amplitudes (in the µV range). With the placement of the EEG electrodes shown in Figure 5f, representative EEG signals are recorded during eye‐opening and eye‐closing states (Figure 5g‐i). A distinct peak at approximately 9 Hz during the eye‐closed state from fast Fourier transform (FFT) analysis (Figure 5g‐ii) corresponds to the alpha (α) rhythm. The alternated eye‐closed and eye‐open states (60 s each, repeated for three cycles) are also captured by the corresponding time–frequency analysis (Figure 5g‐iii). During eye opening, the EEG signals exhibit a broader frequency distribution, accompanied by a marked attenuation of the α rhythm. These results confirm that the LIG–Ag/SEBS electrodes can reliably capture relatively weak EEG signals.

The current demonstrations of 12‐lead ECG monitoring and robotic hand control primarily serve as proof‐of‐concept validations of the proposed LIG–Ag/SEBS epidermal electrodes in controlled laboratory settings. Although the present system demonstrates stable, high‐quality signal acquisition under dynamic deformation, practical deployment of wearable EP systems will require additional considerations beyond material‐level performance. Key challenges include soft‐rigid integration with electronic modules, signal stability under perspiration, cross‐user algorithm model optimization, and other real‐world factors. Future work will therefore focus on improved system integration, adaptive signal processing, larger‐cohort validation, and personalized machine‐learning‐based control strategies for reliable real‐world HMI applications.

## 3 Conclusion

In summary, this study demonstrates a simple and effective strategy to fabricate large‐area epidermal electrodes by leveraging the hydrophilicity of LIG to selectively wet silver ionic solutions, followed by thermal reduction to obtain highly conductive LIG–Ag nanocomposites on an ultrathin SEBS elastomer. The resulting electrodes exhibit excellent conformability, low interfacial contact impedance, and long‐term durability for EP signal monitoring with high SNR and strong resistance against motion artifacts. The proof‐of‐concept demonstrations for 12‐lead ECG monitoring and EMG‐based robotic hand control confirm the suitability of the LIG–Ag/SEBS epidermal electrodes for multi‐channel medical monitoring and HMI applications. At present, the electrode performance has been validated primarily under controlled laboratory conditions, and systematic evaluation across larger subject populations and longer wearing times will be required to fully determine clinical reliability. Future efforts may also focus on integrating the electrodes with fully wireless systems and advanced signal‐processing algorithms to enable scalable, long‐term bioelectronic interfaces for real‐world healthcare and rehabilitation applications.

## 4 Experimental Section

### 4.1 Materials

Commercially available polyimide (PI) sheets were purchased from DuPont (Kapton HN, thickness 60 µm). Water‐soluble tape (ASWT‐1) was obtained from Aquasol. Silver acetate (99%), ammonium hydroxide (28%), and formic acid (97%) were obtained from Alfa Aesar. Ethanolamine (99%) and potassium hydroxide (99.99%, metals basis) were provided by Shanghai Macklin Biochemical Co., Ltd. Polystyrene‐block‐poly(ethylene‐ranbutylene)‐block‐polystyrene (SEBS) powder (M_W_ ≈ 118000 with 21:9 mole ratio of styrene: rubber) was supplied by Sigma–Aldrich. Toluene (99.5%) was made available from General Reagent.

### 4.2 Preparation of Silver Ionic Solution

Silver acetate (4 g) was weighed and added to a brown glass bottle, followed by the addition of 10 mL of ammonium hydroxide. While stirring the mixture on a magnetic stirrer, 1 mL of formic acid was slowly added. The bottle was sealed, and the solution was aged at room temperature for 24 h. The resulting silver ionic solution was then filtered through a 0.22 µm syringe filter. All procedures were carried out in the fume hood.

### 4.3 Fabrication of LIG–Ag Nanocomposite

A layer of water‐soluble adhesive was applied onto a 10 cm × 10 cm glass substrate, and a PI film was subsequently attached to the adhesive layer. The PI film was cleaned with 70% isopropanol and dried using dust‐free paper. Laser‐induced graphene was directly patterned on the PI film using a VLS3.50 Universal laser system. The desired electrode patterns were engraved to form porous LIG, with strong hydrophilicity within 1 h after laser engraving. The laser parameters were set as follows: raster mode, power 4.2% (corresponding to an absolute power of 2.1 W), speed 5.5% (corresponding to an absolute speed of 69.85 mm/s), pulses per inch (PPI) 1000, and image density 6.

The filtered silver ionic solution was drop‐cast onto the LIG patterns using a pipette, leading to rapid, selective wetting on LIG due to its strong hydrophilicity on the near‐hydrophobic PI. The LIG–Ag nanocomposites were obtained by annealing the substrate on a hot plate at 90°C for 10 min, resulting in the in situ formation of Ag nanoparticles within the porous LIG network.

### 4.4 Fabrication of Skin Conformal Electrode

To prepare the SEBS solution, 3 g of SEBS triblock copolymer powder was dissolved in 20 mL of toluene and magnetically stirred until fully dissolved. The resulting SEBS solution was spin‐coated onto the LIG–Ag/PI substrate at 800 rpm for 15 s.

The PI etching solution was prepared by adding 50 mL of deionized water to a beaker, followed by the addition of 30 g of ethanolamine and 20 g of potassium hydroxide. The mixture was stirred until completely dissolved. The SEBS/LIG–Ag/PI structure was then immersed in the etching solution and heated in an 80°C water bath for 2 h to remove the PI layer. After etching, the sample was thoroughly rinsed with deionized water, yielding the ultrathin SEBS/LIG–Ag epidermal electrode.

### 4.5 Characterization

The microstructures of the LIG–Ag nanocomposites were characterized using a scanning electron microscope (SEM; Thermo Scientific Apreo 2C). XRD and Raman spectroscopy were performed using a D8 Advance diffractometer and a LabRAM HR Evolution spectrometer, respectively. Electrical resistance was measured using a digital multimeter (Agilent 3458A). Sheet resistance was measured using a four‐probe sheet resistance meter (HPS58006, Helpass). Skin–electrode contact impedance was evaluated using an electrochemical workstation (CHI660E), with the skin wiped with a wet tissue before testing to ensure consistency.

### 4.6 EP Signal Recording

For all single‐channel EP signal recordings, the epidermal electrodes were conformally attached to human skin, with metal wires connecting the electrodes to a commercialized amplifier (PowerLab 4/26, AD Instruments). ECG signals were recorded with two working electrodes placed on the volunteer's inner wrists and the reference electrode placed on the left foot (sampling rate of 1 kHz with a 1–50 Hz bandpass filter). EMG signals were recorded using two working electrodes with a center‐to‐center distance of 3 cm attached to the forearm, and a reference electrode attached to the elbow joint (sampling rate of 1 kHz, with a 50 Hz notch). Vertical EOG signals were measured with two working electrodes placed on the upper and lower eyelids, respectively, and the reference electrode placed on the forehead. Horizontal EOG signals were measured with two working electrodes placed on the outer canthi of each eye and the reference electrode placed on the forehead. During the measurements, the volunteer was instructed to keep the eyes stationary for 10 s, followed by periodic left–right or up–down movements (sampling rate of 200 Hz, with a 50 Hz notch). EEG signals were monitored with the recording electrode positioned on the left forehead (Fp1) and the ground/reference electrode placed on the right/left mastoids, according to the international 10/20 system (sampling rate of 200 Hz, with a 50 Hz notch). The SNR of the EP signal was calculated as: SNR (in dB) = 10 log $\frac{V_{\mathrm{rmsf}\;}}{V_{\mathrm{noise}}}$, where the subscripts “rmsf” and “noise” represent the RMS values of the filtered signal and noise signal, respectively.

## Conflicts of Interest

The authors declare no conflicts of interest.

## Supporting information




**Supporting File 1**: advs76138‐sup‐0001‐SuppMat.docx.

**Supporting File 2**: advs76138‐sup‐0002‐MovieS1.mp4.

**Supporting File 3**: advs76138‐sup‐0003‐MovieS2.mp4.

## Data Availability

The data that support the findings of this study are available from the corresponding author upon reasonable request.
