# Supplementary material for: Facile Fabrication of Low‐Impedance, Highly Conformal Epidermal Electrodes Based on Laser‐Induced Graphene–Silver Nanocomposites
Source: Adv Sci (Weinh). 2026 Jun 15;13(51):e76138. doi: 10.1002/advs.76138 (PMC13336890; doi:10.1002/advs.76138)
Supplement: Supplementary file 1 [file ADVS-13-e76138-s001.docx]

**Supporting Information**

**Facile fabrication of low-impedance, highly conformal epidermal electrodes based on laser-induced graphene–silver nanocomposites**

Jiuqiang Li^1^, Senhao Zhang^1,3^, Kai Guo^1,2^, Donghai Qiu^1^, Juzhong Zhang^1,2*^, Hongbo Yang^1,2*^, Huanyu Cheng^3*^

^1^Suzhou Institute of Biomedical Engineering and Technology, Chinese Academy of Science, Suzhou, 215011, P.R.China

^2^School of Biomedical Engineering (Suzhou), Division of Life Sciences and Medicine, University of Science and Technology of China, Hefei 230022, P.R. China

^3^Department of Engineering Science and Mechanics, The Pennsylvania State University, University Park, 16802, USA

^*^To whom correspondence should be addressed. E-mail: jzzhang@sibet.ac.cn (**J.Z.**), yanghb@sibet.ac.cn (**H.Y.**), huanyu.cheng@psu.edu (**H.C.**)

**Figure S1.** Contact angles of water on (a) polyimide (PI) and (b) laser-induced graphene (LIG).

**The chemical reactions formation of the active silver precursor:**

$$\text{2}\text{AgCH}_{\text{3}}\text{CO}_{\text{2}}\text{+2}\text{NH}_{\text{4}}\text{OH}\underset{\to}{\text{ H}_{\text{2}}\text{O }}\text{Ag}_{\text{2}}\text{O}\text{+}\text{2}\text{NH}_{\text{4}}\text{CH}_{\text{3}}\text{CO}_{\text{2}}\text{+}\text{H}_{\text{2}}\text{O}$$

$$\text{Ag}_{\text{2}}\text{O+4}\text{NH}_{\text{3}}\text{+2}\text{NH}_{\text{4}}\text{CH}_{\text{3}}\text{CO}_{\text{2}}\text{+}\text{H}_{\text{2}}\text{O}\underset{\to}{\text{ N}\text{H}_{\text{3}}\text{/}\text{H}_{\text{2}}\text{O }}\text{2Ag}{\text{(}\text{NH}_{\text{3}}\text{)}}_{\text{2}}\text{CH}_{\text{3}}\text{CO}_{\text{2}}\text{+2}{\text{N}\text{H}}_{\text{4}}\text{O}\text{H}$$

The final reactive silver ink primarily consists of diamminesilver(I) cations ([Ag(NH_3_)_2_]^+^), along with acetate and formate anions.

**Figure S2.** Photograph of the prepared silver ionic solution.

**Figure S3.** Photographs of electrodes at different fabrication stages: LIG (left), LIG–silver (Ag) (middle), and LIG–Ag/SEBS (right).

**Figure S4.** Scanning electron microscope (SEM) images of LIG–Ag. (a) SEM image showing the widespread distribution of Ag particles on the LIG surface. (b) High-magnification SEM image corresponding to the red dashed box region in (a).

**Figure S5.** Particle size distribution histogram of the deposited Ag particles extracted from high-magnification SEM images.

**Figure S6.** Raman spectra of LIG (black) and LIG–Ag (red).

**Figure S7.** Sheet resistance of LIG as a function of silver deposition densities before (black) and after (red) transfer.

**Figure S8.** Normalized relative resistance changes of the LIG–Ag/SEBS electrode with different silver deposition densities as a function of tensile strain from 0 to 60%.

**Figure S9.** Schematic illustration of the percolation network models of (a) pristine LIG and (b) LIG–Ag electrodes (i) before and (ii) after tensile deformation.

**Figure S10.** Tensile stress-strain curves of SEBS and the SSA composite.

**Figure S11.** Relative resistance change of the LIG–Ag/SEBS epidermal electrode during loading-unloading cycles at tensile strains of 30% (green) and 50% (blue).

**Figure S12.** Long-term cyclic stability of the LIG–Ag/SEBS epidermal electrode under 30% strain.

**Figure S13.** Linear pattern arrays with varying (a) line widths and (b) spacings.

**Figure S14.** Adhesion force versus displacement curves of LIG–Ag/SEBS electrodes sprayed with a liquid bandage.

**Figure S15.** Comparison in ECG signals measured by LIG–Ag/SEBS electrodes with (black) and without (red) liquid bandage.

**Figure S16.** ECG signals and corresponding SNR of LIG–Ag/SEBS electrodes under 30% strain.

**Figure S17.** (a) ECG signals and (b) corresponding SNR comparison of LIG–Ag/SEBS electrodes with a higher Ag deposition density (20 μL cm^-2^).

**Figure S18.** **Skin irritation comparison between the LIG**–**Ag/SEBS and commercial Ag/AgCl electrodes over 24 hours.** (a) Photograph of the LIG–Ag/SEBS electrode (left, yellow rectangle) and a standard commercial Ag/AgCl gel electrode (right, red circle) attached to a human forearm. (b) Skin appearance after electrode removal, showing minimal skin irritation induced by the LIG–Ag/SEBS electrode compared with obvious erythema caused by the commercial Ag/AgCl electrode.

**Figure S19.** Stability of the LIG–Ag/SEBS epidermal electrodes under high-humidity conditions (90% RH). (a) Relative resistance variation during 7 days of exposure. (b) Representative ECG signals recorded before and after humidity exposure, showing negligible degradation in signal quality and SNR.

**Figure S20.** Fabrication process of the precordial LIG–Ag/SEBS electrode array. (a) Pristine PI substrate. (b) Laser-patterned LIG–Ag conductive layout on PI. (c) SEBS-coated LIG–Ag/PI structure. (d) Released ultrathin LIG–Ag/SEBS electrode after PI removal.

**Figure S21.** Custom-built 12-lead ECG acquisition and processing circuit board.

**Table S1.** Cost analysis of LIG–Ag/SEBS electrodes for 12-lead ECG fabrication

| **Component** | **Cost Determination**  **(per sensor)** | **Total** |
| --- | --- | --- |
| PI sheet | $0.1 | $1.6 |
| SEBS (0.5 g) + toluene (3 mL) | $0.2 |  |
| Silver acetate (0.04 g) + ammonium hydroxide (100 μL) + formic acid (10 μL) | $0.5 |  |
| Double-sided tape + water-soluble adhesive | $0.1 |  |
| Ethanolamine (3 g) + potassium hydroxide (2 g) | $0.2 |  |
| Laser processing (VLS3.50 system, amortized) | <$0.5 |  |

**Table S2.** Performance comparison between LIG–Ag/SEBS and previously reported soft epidermal electrodes. The reported SNR values were obtained under specific testing conditions and should be interpreted as indicative of general performance trends rather than absolute or directly comparable rankings.

| **Materials** | **Patterning method** | **Sheet resistance（Ω sq^-1^）** | **Long stability** | | **SNR** | **Ref** |
| --- | --- | --- | --- | --- | --- | --- |
| AgNW/GN/ SEBS | Mask-required | 16.1 | > 1 day | 23.7 ECG | | [1] |
| PEDOT:PSS/ LIG | Mask-required | 13.3 | > 120 days | 12.9 ECG | | [2] |
| PEDOT:PSS/ textile | Mask-required | 5.6 | 36 h | 15.42 ECG | | [3] |
| CNT/PDMS | Unpatterned | 10 | N/A | 14.58 EMG | | [4] |
| PVA/b-PEI | Unpatterned | N/A | No | 8.5 EMG | | [5] |
| PEDOT:PSS/ PSBMA/PVA | Mask-free | N/A | No | 16.26 EMG | | [6] |
| LIG**-**Ni/Dragon Skin | Mask-free | 151.5 | N/A | 15.2 ECG | | [7] |
| AgNWs/TPU | Mask-free | 7.3 | N/A | 7.0 ECG | | [8] |
| **LIG–Ag/SEBS** | **Mask-free** | 0.35 | > 240 days | **18.9 ECG/ 30.3 EMG** | | **This work** |

**References:**

[1]	Y. Liu, B. Xu, Z. Xie, J. Yang, Y. Liu, Y. Yang, H. Xu, ACS Appl. Mater. Interfaces 2023, 15, 59787.

[2]	M. Abu Zahed, P. S. Das, P. Maharjan, S. C. Barman, M. Sharifuzzaman, S. H. Yoon, J. Y. Park, Carbon 2020, 165, 26.

[3]	R. Castrillón, J. J. Pérez, H. Andrade-Caicedo, Biomed. Eng. Online 2018, 17, 38.

[4]	B.-C. Kang, T.-J. Ha, Japanese Journal of Applied Physics 2018, 57, 05GD02.

[5]	Y. Liu, C. Wang, J. Xue, G. Huang, S. Zheng, K. Zhao, J. Huang, Y. Wang, Y. Zhang, T. Yin, Z. Li, Adv. Healthcare Mater. 2022, 11, 2200653.

[6]	J. Yu, R. Wan, F. Tian, J. Cao, W. Wang, Q. Liu, H. Yang, J. Liu, X. Liu, T. Lin, J. Xu, B. Lu, Small 2023, n/a, 2308778.

[7]	Y. Tong, Y. Zhang, B. Bao, X. Hu, J. Li, H. Wu, K. Yang, S. Zhang, H. Yang, K. Guo, Bioengineering 2023, 10, 620.

[8]	W. Zhou, S. Yao, H. Wang, Q. Du, Y. Ma, Y. Zhu, ACS Nano 2020, 14, 5798.
